# Surgical prescription of *epoetin alfa* in contemporary total hip arthroplasty: a prospective comparative study

**DOI:** 10.1007/s00264-019-04399-7

**Published:** 2019-08-29

**Authors:** Hervé Hourlier, Peter Fennema

**Affiliations:** 1 Department of Orthopedic Surgery, Polyclinique de la Thiérache, Rue du Dr Koral, 59212 Wignehies, France; 2 AMR Advanced Medical Research GmbH, Hofenstrasse 89b, 8708 Männedorf, Switzerland

**Keywords:** Arthroplasty, Replacement, Hip, Anaemia, Bleeding index, Blood management, Recombinant erythropoietin therapy, Surgical prescript

## Abstract

**Purpose:**

Pre-operative anaemia treatment has been associated with reduced morbidity in joint arthroplasty. This study examined the impact of a surgical prescription of epoetin (EPO) in contemporary total hip arthroplasty (THA).

**Methods:**

We conducted a comparative study in a series of 1402 primary THAs performed in patients all having a pre-operative haemoglobin (Hb) level documented four to eight  weeks before THA surgery. In group A (647 hips), one subcutaneous injection of 40,000 IU EPO once a week for four weeks was prescribed at the discretion of anaesthetist during the pre-operative visit in patients with pre-operative Hb between 10 and 13 g/dl. In group S comprising the remaining 755 hips, an amended EPO therapy including two  injections of 20,000 to 40,000 IU was prescribed by the surgeon in patients with Hb less than 12 g/dl deemed at high risks to be transfused following THA. Primary study endpoint was the bleeding index (BI).

**Results:**

EPO therapy was delivered in 43 patients (6.7%) in group A and in 26 patients (3.4%) in group S (*p* = 0.006). The mean total dose of EPO administrated was 115,349 IU in group A versus 75,200 IU in group S (*p* < 0.001). The mean BI were 2.7 ± 1.0 in group A and 2.8 ± 1.0 g/dl in group S (*p* = 0.375). No patient was blood-transfused up to post-operative day seven in group S versus five patients in group A (*p* = 0.021).

**Conclusions:**

The amended protocol does not lead to increased peri-operative bleeding. Advances in intra-operative methods to reduce the bleeding allow changing indications of EPO in patients undergoing THA with a low level of Hb.

## Introduction

Pre-operative anaemia is a well-established risk factor for blood transfusion following total hip arthroplasty (THA) [1]. Anaemia has been estimated to affect 15 to 44% of the adult population undergoing total joint arthroplasty (TJA), depending on the definition of anaemia [1, 2]. Pre-operative anaemia is associated with increased morbidity, mortality, and costs in non-cardiac surgery [3] and orthopaedic surgery [4], and should therefore be corrected before surgery [5, 6]. The correction of anaemia prior to THA has been associated with reduced morbidity, hospital stay, and costs [7]. Haemoglobin (Hb) level can be increased before TJA with iron, folates, vitamin B12, and erythropoiesis-stimulating agents such as epoetin (EPO) [7, 8].

EPO is approved by our National Health Authorities for hip surgery, and dosage instructions have not been changed for two decades. EPO is also recommended by the European Medicinal Agency to reduce the need for blood transfusions in adults with moderate anaemia who are about to undergo major orthopaedic (bone) surgery, such as hip surgery. Several studies have confirmed the effectiveness (i.e., by means of the ability to decrease the rate of blood transfusion [5, 6, 9, 10]) as well as the safety [11]. Only few centres reported that they administer EPO to optimize patients before surgery [12]. The decision to use EPO on a routine basis should be balanced against its costs [11]. In addition, the organizational burden of EPO application needs to be considered, as EPO therapy needs to be applied well before surgery.

To date, there is wide consensus in the orthopaedic community to revisit (re-audit) the modalities of EPO therapy. Notably, the routine use of antihaemorrhage antifibrinolytic agents such as tranexamic acid (TXA) [13, 14], the adoption of more restrictive transfusion protocols, and the advances in less invasive THA approaches reduce transfusion and could amend the use of EPO [15].

In 2005, our unit initiated a blood management program that included the use of pharmacologic agents instead of auto transfusion of blood [16, 17]. TXA was used routinely by anaesthetists and EPO therapy was prescribed in accordance with the product’s instructions for use (standardized regimen of one weekly 40,000 IU epoetin alpha injection for 4 weeks) and the national guidelines indications (preoperative Hb between 10 and 13 g/dl). In 2013, we re-audited our EPO plan and some changes were implemented. EPO prescription was conveyed by the surgeon with lowering the indications as well as lowering the total dose of EPO.

The primary aim of this study was to examine the impact of the amended surgical prescription of EPO therapy in contemporary THA. We hypothesised that the amended protocol would not increase peri-operative bleeding in patients undergoing THA.

## Materials and methods

We conducted a retrospective comparative study utilizing prospectively collected data in a single-surgeon series of unilateral primary THAs performed in patients having an Hb level documented four to eight  weeks before THA. Patients who underwent revision arthroplasties, bilateral arthroplasties, and resurfacing arthroplasties and patients with recent fractures of the femur were not included. Furthermore, patients receiving heparin at a therapeutic dose before the surgery were excluded. In group A (647 hips), a standardized conservative regimen of EPO was prescribed at the discretion of anaesthetist during the pre-operative anaesthetist visit in patients with Hb between 10 and 13 g/dl considered at risk to receive blood transfusion following THA. That regimen consisted of one subcutaneous injection of 40,000 IU Epoetin alpha (Eprex, Janssen, Issy-les-Moulineaux, France) once a week for four  weeks before surgery. In group S, comprising the remaining 755 hips, EPO therapy was prescribed by the surgeon via a dose of 20,000 to 40,000 UI epoetin alpha injected at 14 days and seven  days before surgery in patients without uncontrolled arterial hypertension having an Hb less than 12 g/dl and deemed at high risk to be transfused following THA. EPO was injected subcutaneously at the patient’s home by a nurse. EPO was administered in combination with oral iron supplementation (Tardyferon, Pierre Fabre, Castres, France) (240 mg daily), except in patients who declared to be intolerant to oral iron. The latter patients received intravenous (IV) iron (Ferinject, Vifor Pharma, Paris, France). The quality of delivery was recorded in the patient file.

In the majority of patients (*n* = 1188, 84.7%), one single TXA bolus of 20 to 30 mg/kg was administered after induction of anaesthesia [18]. The contraindications of TXA were a venous or arterial thromboembolic event in the previous 3 months. TXA was not given to patients who were not deemed at risk for post-operative blood transfusion.

On the day of surgery, pharmacological venous thromboembolism prophylaxis was initiated via 10 mg oral rivaroxaban (Xarelto, Bayer, Lille, France) given six to eight hours  after closure. This dose was repeated daily for 35 days without platelet monitoring. The administration of another anticoagulant drug was avoided [19].

In all hips, a cementless implant was implanted via a minimally invasive anterolateral approach in a lateral position [20]. Blood salvage, a drain system or local infiltration analgesia were not used during the course of the study. Peri-operative protocols included multimodal pain management and standard antibiotic regimens.

In this study, only venous blood samples were used to determine Hb levels, which were controlled at admission (post-operative day − 1 (POD-1)), at POD1, and POD7 ± 1. In cases where the patient was discharged before POD7, Hb was controlled with blood collected at home on POD7. A Doppler ultrasound of both lower limbs was performed at discharge on POD7, or earlier in cases where deep vein thrombosis (DVT) was suspected. Patients who were considered to be at risk of cardiovascular complications were monitored for troponin elevation post-operatively. All complications were recorded up until 90 days. The following events were defined as major complications: death, cerebrovascular accident, peri-operative myocardial infarction, symptomatic pulmonary embolism, and proximal DVT. Primary endpoint was the bleeding index (BI). For each patient, BI was calculated by subtracting the Hb level at POD-1 from the Hb level at POD7, and then adding the number of units of packed red blood cells (PRBCs) transfused within that timeframe, under the assumption that the transfusion of one PBRC raises Hb by 1 g/dl [21, 22]. Total blood loss was estimated using a standardized method [23]. Blood transfusion was administered at a Hb threshold of 7 g/dl in patients without comorbidities, and at a threshold of 8 g/dl in patients with anaemia symptoms or with pre-existing cardiac insufficiency or coronary heart disease. Total blood loss and transfusion were the secondary study endpoints.

All patients provided written informed consent prior to study commencement and no patients in the present study declared religious convictions declared against blood transfusion. In accordance with French law, ethics committee approval was not obtained, as the study was purely observational, with no changes to standard clinical practice.

### Statistical analysis

Baseline data are presented as mean ± standard deviation (SD) for continuous variables, and as counts and percentages for categorical variables. The baseline differences among patients in group A and group S were analysed with use of the Fisher’s exact or the Pearson’s chi-squared test for categorical variables, the Mann-Whitney test for ordinal variables, and the two samples *t* test for continuous variables. *p* values < 0.05 were considered significant. Stata (Stata Corp, College Station, TX) version 15.1 was used for all statistical analyses.

## Results

The two study groups were comparable in terms of age and sex and pre-operative Hb level, but there were, comparably, slightly more patients in Group A with ASA 3 and there were minor differences in terms of mean cell volume (Table 1). EPO therapy was delivered in 43 patients (6.7%) in group A versus 26 patients (3.4%) in group S (*p* = 0.006). The mean ± SD total dose of EPO administrated was 115,349 ± 31.724 IU in group A versus 75,200 ± 13.267 IU in group S (*p* < 0.001). In the present series, a single patient intolerant to oral iron opted for IV iron, while the remainder received oral iron supplementation. The mean ± SD pre-operative level of Hb in patients receiving EPO was 11.2 ± 0.8 g/dl in group A and 11.5 ± 0.9 g/dl in group S (*p* = 0.090). In patients treated with EPO, the average Hb level increased pre-operatively with 2.2 ± 1.1 g/dl in group A and with 1.1 ± 0.9 g/dl in group S (*p* < 0.001).

**Table 1:** Baseline characteristics

	Group A (*n* = 647)	Group S (*n* = 755)	*p* value
Age (years)	69.1 ± 10.6	70.1 ± 10.2	0.097
Sex (female)*	348 (53.8)	383 (50.9)	0.275
BMI (kg/m^2^)	29.8 ± 5.6	29.6 ± 5.3	0.343
ASA class*			0.009
- ASA 1 -ASA 2 - ASA 3 - ASA 4	121 (18.7) 405 (62.6) 119 (18.4) 2 (0.3)	146 (19.3) 525 (69.5) 84 (11.1) 0 (0.0)	
Haemoglobin (g/dl)			
Preoperative:	13.9 ± 1.3	13.9 ± 1.3	0.498
POD − 1	14.0 ± 1.1	13.8 ± 1.3	0.002
Mean cell volume (μm^3^)	92 ± 5	94 ± 5	< 0.001
EPO therapy	43 (6.7%)	26 (3.4%)	*p* = 0.006
Total dose of EPO (IU)	116,000	77,000	*p* < 0.001
Pre-incision tranexamic acid*	511 (79.0%)	677 (89.7%)	< 0.001
Surgical time (minutes)	60 ± 11	60 ± 10	0.430
eGFR (ml//min/1.73/m^2^)	78 ± 31	77 ± 28	0.938

Presented as mean ± standard deviation, except * presented as *n* (%). *ASA* American Society of Anesthesiologists Classification, *BMI* body mass index, *eGFR* estimated glomerular filtration rate, *EPO* epoietin, *POD* postoperative day

No significant differences were observed in terms of the BI (Table 2). During the first peri-operative week, five  patients (0.8%) were blood transfused in group A versus no patients in group S (*p* = 0.021). None of the patients who received blood transfusion had received EPO prior to THA. A significant difference in BI was found between patients treated and not treated with EPO (2.2 ± 1.0 in the EPO group versus 2.8 ± 1.0 in the total group (*p* < 0.001). No adverse events related to the EPO therapy emerged. No major thromboembolic or bleeding-related events in any of the groups were observed.

**Table 2:** Study outcomes

	Group A (*n* = 647)	Group S (*n* = 755)	*p* value
Haemoglobin [g/dl]			
POD 1	12.2 ± 1.2	12.2 ± 1.3	0.696
POD 7	11.3 ± 1.2	11.0 ± 1.4	< 0.001
Bleeding index	2.7 ± 1.0	2.8 ± 1.0	0.375
Estimated total blood loss up to POD7	1001 ± 472	1047 ± 447	0.081
Blood transfusion up to POD7 *	5 (0.8%)	0 (0.0%)	0.021

Presented as mean ± standard deviation, except * presented as *n* (%). *POD* post-operative day

## Discussion

This clinical study expands on our previously pragmatic conducted research on patient blood management, with the aim to minimize blood transfusion and the occurrence of post-operative morbidity due to deleterious anaemia in THA. The present study shows that cessation of blood transfusion can be attained in contemporary THA performed with the detection of pre-operative anaemia and the use of an amended EPO regimen given to anaemic patients before the surgery. To our knowledge, the present series is the first to report the results of amended EPO therapy using both the indications and the regimen proposed by Pierson [24] and by Rosencher [25] respectively. Our results confirm the conclusive algorithm proposed by Pierson, recommending that EPO only be given to patients with preoperative Hb less than 12 g/dl [24]. Notably, the algorithm of Pierson was constructed without gender discrimination by using an equation that includes the pre-operative Hb minus expected blood loss plus the standard deviation of expected blood loss. Pierson considered an historical decline in Hb of 4.1 g/dl, combined with an Hb transfusion trigger of 7 g/dl for the construct of his algorithm. Today, the historical post-operative Hb drift of 4.1 g/dl may be seen as too high because of the routine administration of TXA and the advances in surgical techniques. On the other hand, the transfusion trigger of 7 g/dl has not been widely adopted in most hospitals, since most clinicians use a transfusion threshold of 8 g/dl for patients with anaemia symptoms or with cardiovascular diseases.

To our knowledge, this series is the first to report a zero rate of blood transfusion in a consecutive series of patients with pre-operative Hb less than 12 g receiving only two injections of EPO-alfa. The informational materials for recombinant human EPO stipulated a standardized regimen with four injections of 600 UI/kg each, once per week for four weeks before the surgery. However, a previous study by Rosencher found a regimen of two injections of 40,000 UI EPO to be effective in providing a haematocrit above 40% in the majority of patients [25]. In the current study, the EPO therapy regimen of two pre-operative injections of EPO increased the admission Hb by 1.1 g/dl. This gain was sufficient since no patient was blood transfused.

The regimen of two injections (rather than four) has several advantages: it is less expensive, and it is more convenient for the patient. In addition, it prevents the clinical risks related to an excessive increase in Hb by overdose of EPO. In clinical practice, the time for the delivery of care (2 weeks before the surgery) is convenient to avoid having to postpone surgery.

In the present series, all surgeries were performed by a surgeon involved in the development of a patient blood management program. To our knowledge, the present series is the first reporting the results obtained with a surgical prescription of EPO.

No patients who received EPO were transfused. We found that patients who received EPO had a significantly lower BI, which may be caused by ongoing increased erythropoiesis early in the post-operative course as a result of preoperative EPO therapy [26]. Because the effect of EPO is dose-dependent and, overall, the BI found in the present series was very low, we consider that patients in the present series could have received a too high a dose of EPO than is cost-effective (i.e., a lower dose than the dose of 80,000 IU they received could be given with possibly the same effectiveness).

In the current series, EPO therapy was prescribed by the surgeon. In many European medical institutions, EPO is typically prescribed during the anaesthesia visit, but the effectiveness of this practice may not be optimal in terms of percentage of patients treated, mainly for logistical reasons [4]. For example, Kotzé et al. reported 29% of their study population were to be prescribed with EPO, but were untreated for organizational reasons [4]. Bedair reported only 30% of eligible patients accepted EPO treatment [5]. For Thuesinger et al., a major obstacle in the treatment of pre-operative anaemia is that the patient’s surgery is generally scheduled within four weeks. According to the authors, this leaves little time to correct anaemia [27]. In the current series, the forward-thinking original decision to write the prescription of EPO therapy at the surgical visit (instead of the anaesthesia visit) in accordance with the date of operation resulted in a full acceptance and compliance of patients.

The use of EPO was reported to be more effective when combined with IV iron [28]. In the current series, this alternate, but more costly, approach was proposed at the surgical visit to patients who declared to be intolerant to oral iron. However, only one patient in the current series opted for this therapeutic option.

Based on the clinical information provided by this internal audit study, we decided in 2017 to modify our EPO therapy plan with the aim to optimize its cost-effectiveness. We nowadays reserve EPO for patients with a preoperative Hb level of less than 11.5 g/dl. The period to detect pre-operative anaemia is three  weeks (instead of four) before surgery, and we use a dose of 20,000 UI by injection (instead of 40,000 IU) two  weeks and one week pre-operatively. Preliminary results with this new protocol are encouraging.

Surgeons should use the most cost-effective dose when they are considering the use of EPO in anaemic patients undergoing THA. By using 20,000 IU instead of 40,000 UI dose, and by administering two rather than four injections, the costs of our current EPO plan are four-fold less compared to the conventional regimen. Since to date our indications for EPO are limited to patients with preoperative Hb < 11.5, we consider that EPO therapy has become a niche in contemporary THA.

Our study must be interpreted in light of its limitations. This study is not randomized and included a relatively small number of patients presenting with pre-operative anaemia. The two study groups were not perfectly balanced in terms of baseline characteristics. A formal cost-effectiveness assessment of EPO was not performed. However, an economic evaluation was not the purpose of the study and we used EPO with relative parsimony via a restrictive protocol including the lowest critical Hb value reported in literature (12 g/dl) and a regimen of two injections instead of four. To date, one dose of 40,000 IU epoetin alpha can be obtained for less than 70 euros. Another limitation relates to the observational nature of the study, which carries with it a degree of unmeasured, unknown, and confounding factors. Strengths of the study include the homogeneous cohort of patients with primary unilateral THA operated on by a single high-volume surgeon and senior anaesthetist team with perioperative protocols intended to minimize the transfusion rate. Another strength is that all data were prospectively collected. Because Hb levels are usually the main criteria for pharmacological optimization and for transfusion in THA, the Hb concentrations were controlled at pre-defined time points. As the formula currently used to calculate blood loss does not ensure that the values obtained are sufficiently accurate for absolute measurement [29], we use an index we developed ten  years ago, which takes into account the impact of transfusion in the decline of Hb for the peri-operative period of 7 days [19, 22]. This interval allows us to eliminate the biases due to the decrease of Hb following THA surgery.

Advances in intra-operative methods to reduce the bleeding allow changing indications and posology of EPO in patients undergoing contemporary THA with a low level of Hb. In our view, EPO remains an essential medicine to reduce morbidity related to anaemia. EPO therapy seems a viable treatment option to optimize peri-operative Hb levels and to achieve effective cessation of blood transfusion in patients with a low pre-operative Hb concentration without iron deficiency. The use of two EPO injections (versus four) in contemporary THA seems to be sufficient. A (near) zero rate of blood transfusion may be a new benchmark for arthroplasty. Further studies are required to assess the optimal dosage and indications of EPO as well to confirm the generalizability of our study findings.
